# Exploratory metatranscriptomic survey of bat-associated RNA viruses in Quzhou city, China

**DOI:** 10.3389/fvets.2026.1721079

**Published:** 2026-07-10

**Authors:** Qing Gao, Yide Hu, Chunfu Fang, Guoping Cao, Shuangqing Wang, Conghua Ji, Zhenyu Gong, Bingdong Zhan

**Affiliations:** 1 School of Public Health, Zhejiang Chinese Medical University, Hangzhou, Zhejiang, China; 2 Infectious Disease Control Department, Taizhou Center for Disease Control and Prevention, Taizhou, Zhejiang, China; 3 Infectious Disease Control Department, Quzhou Center for Disease Control and Prevention, Quzhou, Zhejiang, China; 4 Department of Communicable Disease Control and Prevention, Zhejiang Provincial Center for Disease Control and Prevention, Hangzhou, Zhejiang, China

**Keywords:** bats, epidemiology, high-throughput sequencing, phylogenetics, RNA viruses

## Abstract

Bats are recognized as important reservoirs of diverse RNA viruses, yet the bat virome in Quzhou City, Zhejiang Province, remains poorly characterized. Here, we conducted an exploratory metatranscriptomic survey of bat-associated RNA viruses in this region, with particular attention to viral families that include known zoonotic members. Bats were sampled between August and October 2023 from one roost in each of six administrative areas of Quzhou City. Species identification was performed by Cytb sequencing, and pooled intestinal, lung, and other visceral tissues were subjected to high-throughput sequencing, sequence annotation, community comparison, and phylogenetic analysis. A total of 167 bats were collected, representing *Hipposideros armiger* and *Rhinolophus sinicus*, and 35 pooled libraries were generated. Viral contigs were assigned to seven RNA viral families: *Coronaviridae, Flaviviridae, Astroviridae, Hepeviridae, Hantaviridae, Sedoreoviridae*, and *Arenaviridae. Coronaviridae* was the most frequently detected family (97.14% of pooled libraries), whereas *Sedoreoviridae* was the least frequent (2.86%). The intestinal pool from Kecheng District (1A) showed the greatest family-level diversity, with five viral families detected. PCoA and PERMANOVA indicated significant regional structuring of viral composition (*R*^2^ = 0.315, *P* < 0.001), whereas tissue-type differences were not statistically significant (*R*^2^ = 0.078, *P* = 0.195). Because host species and sampling region were fully confounded in this design, species-level patterns were interpreted descriptively only and not as independent species effects. Phylogenetic analyses identified several genetically divergent contigs, including *Mamastrovirus*-related sequences and multiple *arenavirid* contigs forming distinct branches. However, given the pooled design and the absence of independent laboratory validation, these findings should be interpreted as baseline evidence of viral diversity and geographic structuring rather than confirmation of host association, zoonotic potential, or pathogenicity. This study provides an initial virome profile for batsin Quzhou City and a foundation for future individual-level validation, genome completion, and ecological investigation.

## Introduction

In recent years, the frequency of emerging infectious diseases (EIDs) outbreaks has increased significantly, affecting both quality of life and overall economic development (1). It is estimated that 75% of human EIDs are zoonotic, and among the 1,477 known human infectious disease pathogens, 55% are zoonotic pathogens (2). Most RNA viruses are transmitted from wildlife to humans through cross-species transmission. To date, over 224 RNA viruses have been found to cause human diseases, of which 88% are essentially zoonotic (3). Recent EID outbreaks have been largely driven by zoonotic viruses, including Severe Acute Respiratory Syndrome Coronavirus (SARS-CoV), Middle East Respiratory Syndrome Coronavirus (MERS-CoV), Severe Acute Respiratory Syndrome Coronavirus 2 (SARS-CoV-2), and Monkeypox virus (4–6).

Studies have identified bats as one of the most important natural reservoirs of known zoonotic viruses (7). With the expansion of global travel, trade, urbanization, and associated land-use changes, human contact with wildlife such as bats has increased, raising the risk of viral spillover from animals to humans and consequently increasing the likelihood of epidemic outbreaks (8).

As one of the most species-rich groups of mammals, bats comprise 1,473 known species across 21 families and 236 genera worldwide, accounting for approximately 20% of all mammalian species (9). Bats can host diverse viruses while often showing limited clinical disease, which has been linked to unique antiviral and immune-tolerance features, including modulation of inflammatory pathways (e.g., dampened NOD-like receptor thermal protein domain associated protein 3 inflammasome activation) and distinctive interferon biology (10). However, immune tolerance does not imply higher zoonotic risk *per se*; spillover depends on contact networks, exposure pathways, viral determinants of host range, and environmental context (11). Their unique biological, physiological, and immunological traits—such as long-distance migration, extended lifespans, and large-scale social roosting behavior (12, 13)—significantly enhance their viral carriage capacity and the risk of cross-species transmission. Since rabies virus was first detected in asymptomatic bats in 1955 (14), various severe zoonotic viruses have been identified in different bat species and regions, capable of causing serious epidemics or endemics in human populations (15). It has been reported that viral shedding in bats coincides with peak periods of cross-species transmission to humans and other animals, making bat-borne viruses a primary cause of many recent EIDs via diverse transmission mechanisms (16).

Quzhou City, located in western Zhejiang Province, is recognized as a central hub at the intersection of four provinces, bordering Anhui, Jiangxi, and Fujian. The region is characterized predominantly by mountainous and hilly terrain and features a typical subtropical monsoon climate with distinct seasonal variation. These environmental conditions foster rich biodiversity and make the region a potential hotspot for the transmission of zoonotic diseases. To effectively respond to emerging and re-emerging zoonotic diseases, this study conducted bat sampling across six counties (cities and districts) in Quzhou. Using high-throughput sequencing (HTS) technology, we analyzed the diversity of viruses present, their genetic characteristics, and the associated ecological influencing factors. This study provides baseline data on the diversity and phylogenetic composition of bat-associated RNA viruses in Quzhou City and offers a foundation for future surveillance, individual-level validation, and ecological investigation.

## Materials and methods

### Specimens and sampling sites

**Figure 1:**
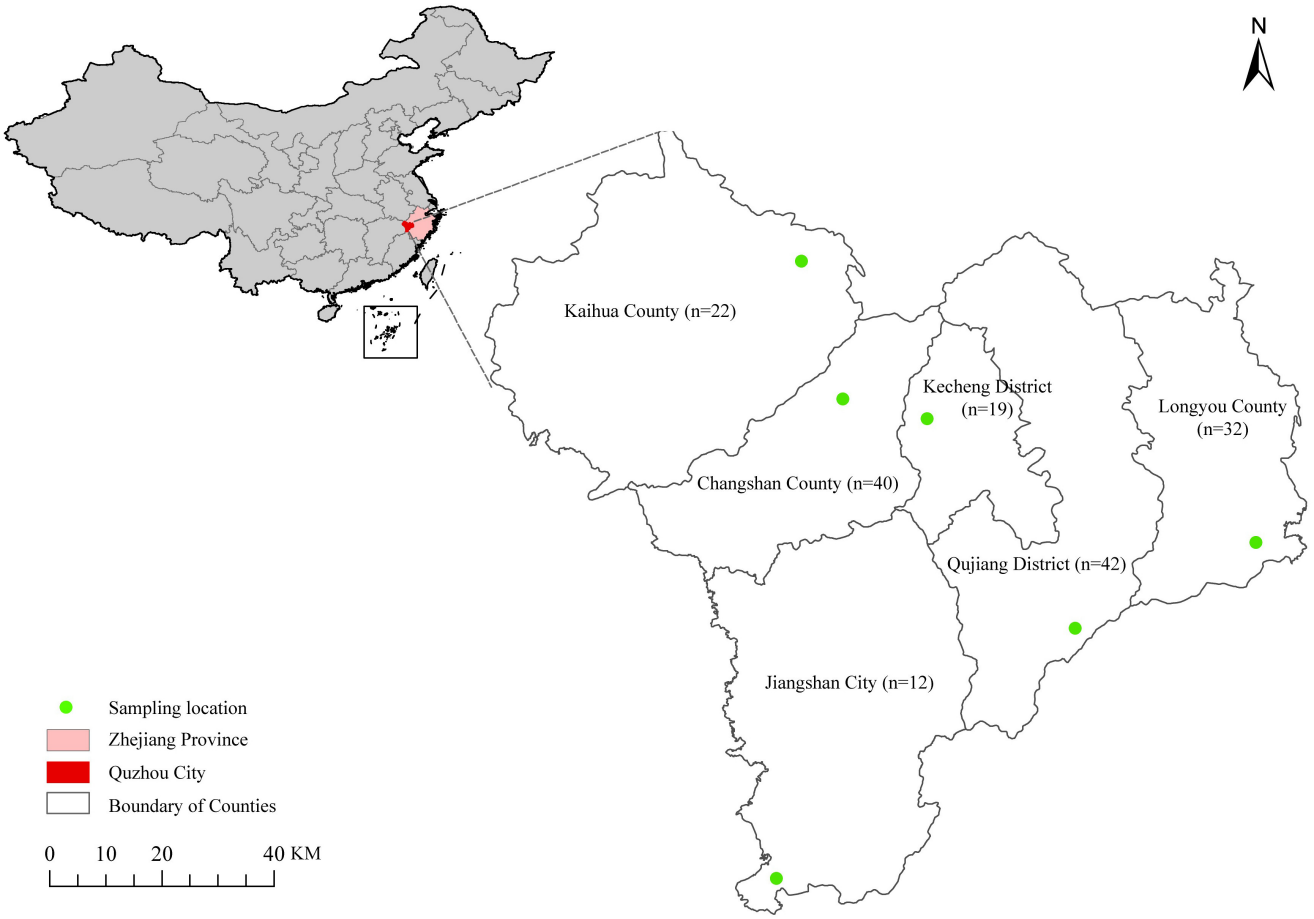
Distribution map of sampling locations.

To investigate the diversity and phylogenetic composition of bat-associated RNA viruses, field surveys were conducted between August and October 2023 in six administrative divisions (counties, cities, or districts) in Quzhou City, Zhejiang Province. Bat roosting sites near areas of human activity, including caves and abandoned buildings, were randomly selected (Figure 1). A total of 167 apparently healthy bats were captured with efforts to minimal disturbance to local bat populations and their habitats.

### Sample collection and processing

Different capture tools, including hand nets and mist nets, were used based on habitat type. Field personnel wore level II biosafety protection, including protective clothing, gloves, masks, and face shields. Environmental metadata including Global Positioning System (GPS) location and weather conditions were recorded at each site.

Bats were humanely euthanized using ether anesthesia followed by exsanguination or cervical dislocation under deep anesthesia, ensuring minimal suffering. All dissections were performed under biosafety level II conditions, and dissection surfaces and tools were disinfected with 75% ethanol before each procedure to prevent cross-contamination. Muscle tissues were collected for mitochondrial cytochrome b (Cytb) amplification and species identification. Lungs, heart, liver, spleen, kidneys, and intestines (including feces) were collected for metagenomic analysis of zoonotic viruses.

Tissue samples were pooled based on sampling location, tissue type, and sample availability. Lung and intestinal samples were analyzed separately due to their direct exposure to the external environment. For each region: (1) one intestinal pooled library, (2) two lung pooled library, and (3) three pooled library from other internal organs (heart, liver, spleen, kidneys) were prepared, except in Jiangshan City, where only two pooled libraries were available for the other-internal-organs category owing to limited sample numbers. These libraries should be regarded as independent pooled libraries rather than technical replicates. This pooling design was intended for cost-effective viral discovery and community-level comparison rather than estimating individual-level prevalence, coinfection, or tissue tropism. All pooled samples were stored at −80 °C for further analysis.

### Species identification

Muscle tissues were used for Cytb gene amplification and sequencing to identify bat species (For detailed information, please refer to the Supplementary materials).

### RNA extraction, library construction, and sequencing

Total RNA was extracted from pooled samples using the QIAGEN AllPrep Power Viral RNAKit (QIAGEN, Hilden, Germany). RNA quantity was assessed using a micro-spectrophotometer. After rRNA depletion, purified and amplified RNA was used to construct pooled sequencing libraries. Sequencing was performed on the DNBSEQ-T7 platform using paired-end 150 bp reads.

### Bioinformatic analyses

The raw sequencing data were first processed with fastp version 0.23.2 (HaploX, China) to remove adapter sequences and low-quality bases. Subsequently, Bowtie2 v2.4.5 (Langmead Lab, Johns Hopkins University, Baltimore, MD, USA) was used to align the reads to the host reference genomes (*Hipposideros armiger*: GCF_001890085.2; *Rhinolophus sinicus*: GCA_001888835.1), and host-derived sequences were filtered out. The clean reads obtained after quality control were *de novo* assembled using MetaSPAdes in meta mode to generate contigs, and only sequences with a length ≥1 kb were retained for downstream analysis.

To identify candidate viral sequences, contigs were first aligned against the NCBI nr database using DIAMOND v2.0.15 blastx with an E-value cutoff of 1e-5, and those matching entries under taxon ID=10239 (viral taxa) were preliminarily selected. A filter list comprising plant, fungal, bacterial, and archaeal viruses, as well as retroviruses, was compiled based on information from Virus-Host DB. DIAMOND blastx (E-value ≤ 1e-5) was reapplied to screen and remove sequences matching these taxa, thereby obtaining higher-confidence viral candidates.

To further focus on RNA viruses, a key gene—RNA-dependent RNA polymerase (RdRp)—was targeted. All RefSeq-recorded RdRp protein sequences were downloaded to construct a custom database. Candidate contigs were aligned against this database using DIAMOND blastx (E-value ≤ 1e-5), and sequences matching RdRp were retained. These sequences were then realigned against the nr database; those whose top hit was not a viral protein were considered false positives and removed. The filtered candidate contigs were subjected to open reading frame (ORF) prediction using ORFfinder version 0.4.3 (National Center for Biotechnology Information, Bethesda, MD, USA), and the corresponding amino acid sequences were extracted. These protein sequences were realigned against the nr database using DIAMOND blastp (E-value ≤ 1e-5), and those with a non-viral protein as the top hit were discarded. The remaining sequences were considered final candidate RNA viral sequences.

For community analyses, we summarized contigs assigned to seven viral families detected in this study (*Coronaviridae, Flaviviridae, Astroviridae, Hepeviridae, Hantaviridae, Sedoreoviridae*, and *Arenaviridae*). These families were retained because they were recovered by the analysis pipeline and include members that have been discussed in zoonotic or wildlife-virus research, but detections were interpreted as bat-associated viral contigs rather than as confirmed zoonotic viruses.

Relative abundances were calculated using Operational Taxonomic Units (OTUs). Beta diversity was assessed using Bray-Curtis distances and visualized by principal coordinate analysis (PCoA). PERMANOVA was used to test tissue-type and regional effects. Because host species and geographic region were completely confounded in this sampling design, species-level ordination was treated as descriptive only and was not interpreted as an independent inferential factor.

Sample similarities were further assessed using Bray–Curtis distances and Ward clustering and a heatmap was generated with the R package (R Foundation for Statistical Computing, Vienna, Austria) pheatmap.

For phylogenetic analysis, reference sequences from the ICTV database were combined with the candidate RNA viral sequences identified in this study. Sequences were aligned using MAFFT version 7.450 (Osaka University, Osaka, Japan), and trimmed with trimAl v1.4.rev22 (Centre for Genomic Regulation, Barcelona, Spain). Maximum likelihood phylogenetic tree was constructed using IQ-TREE v1.6.12 (Center for Integrative Bioinformatics Vienna, Vienna, Austria). The substitution model was automatically selected by the software using the “-m MFP” option. Branch support values were assessed with 1,000 replicates of the SH-like approximate likelihood ratio test (SH-aLRT). All phylogenetic trees were visualized using FigTree v1.4.4 (Institute of Evolutionary Biology, University of Edinburgh, Edinburgh, UK).

## Results

### Bat sample information

Between August and October 2023, bats were sampled from six roosting sites in six counties, cities, or districts of Quzhou City, Zhejiang Province. Among the six sites, the Jiangshan City site was located in a residential area, whereas the remaining five sites were wild caves. A total of 167 bats were collected, including 42 from Qujiang District, 40 from Changshan County, 32 from Longyou County, 22 from Kaihua County, 19 from Kecheng District, and 12 from Jiangshan City.

All bats were identified by morphology and by Cytb sequencing. The samples belonged to two bat families, *Hipposideridae* and *Rhinolophidae*. All bats sampled from Kecheng District, Qujiang District, and Jiangshan City were identified as *Hipposideros armiger* (Har), whereas all bats sampled from Longyou County, Changshan County, and Kaihua County were identified as *Rhinolophus sinicus* (Rsi). Thus, host species and geographic region were fully confounded in the present dataset.

Using the pooling strategy described above, the 167 bat samples were combined into 35 pooled samples, which were used to generate 35 metatranscriptomic libraries. Detailed pooled sample distribution information is provided in Table 1.

**Table 1:** Pooled sample distribution information.

Region	Species	Number	Intestinal tissue	Other visceral tissues			Lung tissue	
Kecheng district	Har	19	1A	1B1	1B2	1B3	1C1	1C2
Qujiang district	Har	42	2A	2B1	2B2	2B3	2C1	2C2
Longyou county	Rsi	32	3A	3B1	3B2	3B3	3C1	3C2
Changshan county	Rsi	40	4A	4B1	4B2	4B3	4C1	4C2
Jiangshan city	Har	12	5A-1	5B1	5B2	-	5C1	5C2
Kaihua county	Rsi	22	6A	6B1	6B2	6B3	6C1	6C2

The first number in the sample number represents the region (1 = Kecheng district, 2 = Qujiang district, 3 = Longyou county, 4 = Changshan county, 5 = Jiangshan city, 6 = Kaihua county), the letter represents the tissue type (A = intestinal tissue, B = other visceral tissue, C = lung tissue), the second number distinguishes independently prepared pooled libraries within the same region and tissue category.

### RNA virome sequencing results

A total of 35 metatranscriptomic pooled samples were subjected to high-throughput sequencing, yielding 775.1 Gb of raw data, with an average of 22.76 Gb per pooled sample. After quality control procedures that removed adapter sequences and low-quality reads, 717.12 Gb of clean data were obtained, with an average of 20.48 Gb per pooled sample. In addition, host genomic sequences and bacterial/fungal-related sequences were further removed. Finally, the remaining reads were assembled by MetaSPAdes, resulting in a total of 9,544,498 contigs. An average of 272,700 contigs were per pooled sample.

### Annotation of RNA virome

After annotation against the NCBI Virus nr database and taxonomic assignment based on ICTV information, viral contigs in this study were assigned to seven RNA viral families: *Coronaviridae* (*n* = 11), *Flaviviridae* (*n* = 22), *Astroviridae* (*n* = 7), *Hepeviridae* (*n* = 8), *Hantaviridae* (*n* = 19), *Sedoreoviridae* (*n* = 5), and *Arenaviridae* (*n* = 28). These counts refer to candidate viral contigs detected in pooled bat-associated samples and do not by themselves establish host status, pathogenicity, or zoonotic relevance.

### Family-level distribution of bat-associated viral contigs

**Figure 2:**
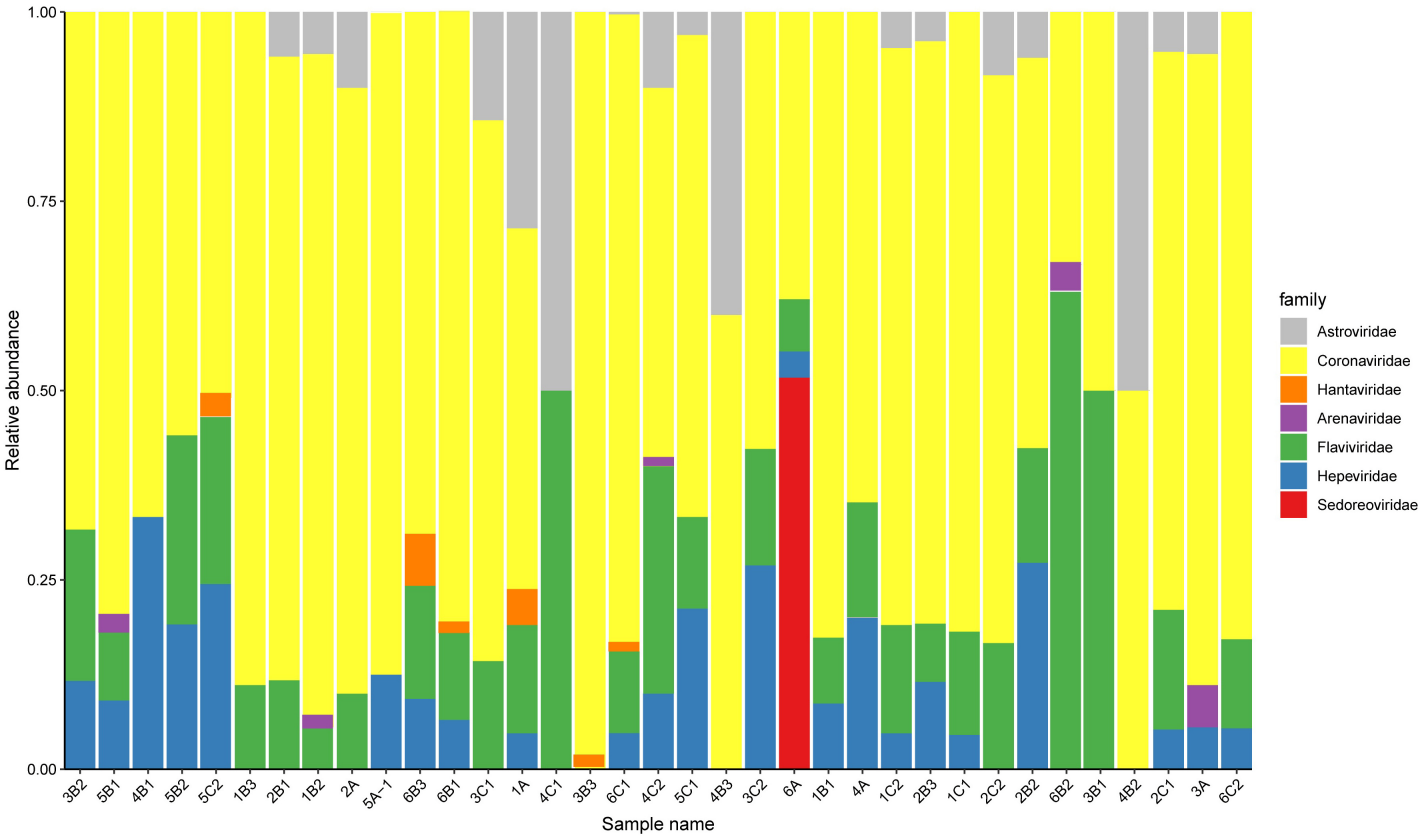
Relative abundance of zoonotic virus families in each sample.

The family-level distribution of viral contigs across the 35 metatranscriptomic samples is shown in Figure 2. *Coronaviridae* was the most widely distributed family and was found in all samples except for the lung group from Changshan County (4C1). The highest relative abundance of *Coronaviridae* was found in the “other internal organs” group from Longyou County (3B3, 97.6%), followed by Kecheng District “other internal organs” groups (1B2 and 1B3, both 88.9%).

*Flaviviridae* was detected in 28 pooled libraries, the highest relative abundance in the “other internal organs” group from Kaihua County (6B2, 66.7%), followed by the “other internal organs” group from Longyou County (3B1) and the lung group from Changshan County (4C1), both with a relative abundance of 50.0%.

*Hepeviridae* was found in 23 samples. The highest relative abundance was observed in the “other internal organs” group from Changshan County (4B1, 33.3%), followed by the “other internal organs” group from Qujiang District (2B2, 27.3%) and the lung group from Longyou County (3C2, 26.9%).

*Astroviridae* was present in 17 samples, with the highest relative abundances in the lung group from Changshan County (4C1) and the “other internal organs” group from Changshan County (4B2), both at 50.0%, followed by 40.0% in 4B3 and 28.6% in the intestinal group from Kecheng District (1A). *Sedoreoviridae* was detected only in the intestinal group sample from Kaihua County (6A), with a relative abundance of 51.7%.

At the family level, the intestinal pooled library from Kecheng District (1A) showed the greatest viral-family diversity, with *Coronaviridae, Astroviridae, Flaviviridae, Hepeviridae*, and *Hantaviridae* all detected. Thirteen pooled libraries contained four viral families (37.1%), 13 contained three viral families (37.1%), and 8 contained two viral families (22.9%).

### Structure and composition of viral communities

**Figure 3:**
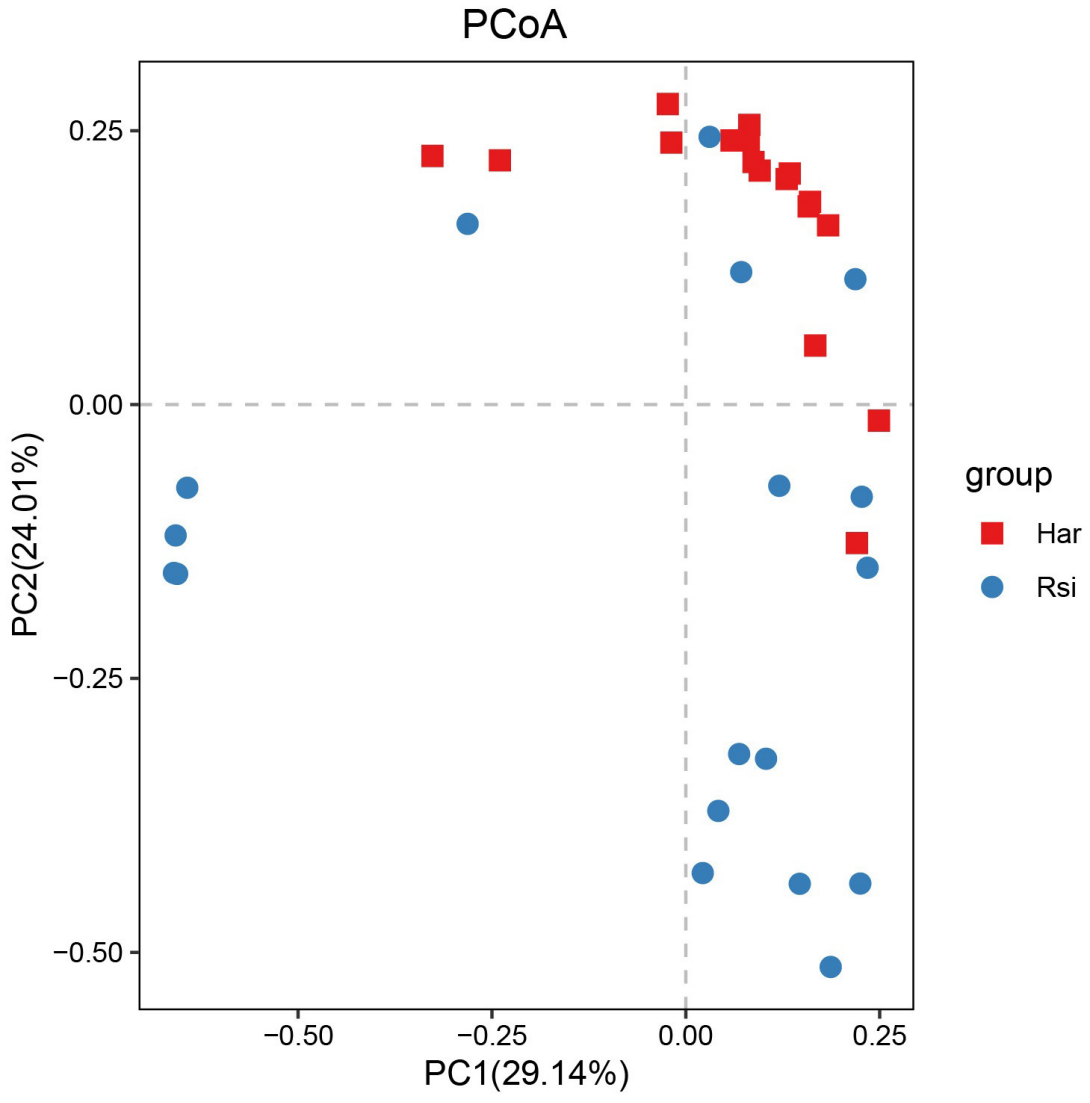
PCoA of pooled libraries grouped by host species.

PCoA based on Bray-Curtis distances was used to summarize differences in viral composition among pooled libraries. Because host species and geographic region were fully confounded, species-based ordination (Figure 3) is presented only as a descriptive visualization and is not interpreted as an independent species effect. Samples assigned to Har and Rsi showed partial overlap in ordination space, and no clear separation attributable solely to species can be inferred from this design.

**Figure 4:**
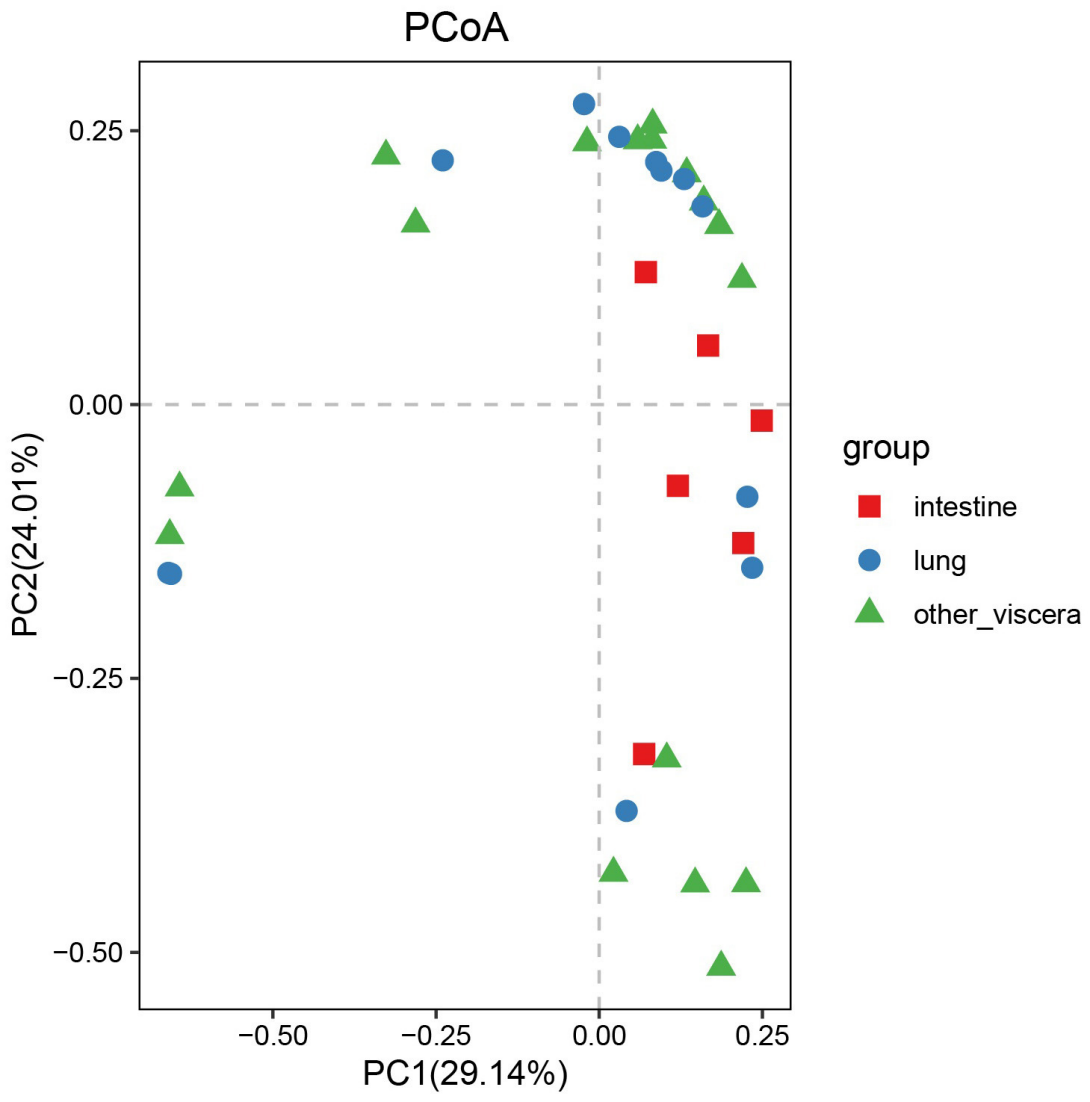
PCoA of pooled libraries grouped by tissue type.

PCoA analysis of intestinal, lung, and other visceral sample types (Figure 4) showed PC1 and PC2 explained 29.14 and 24.01% of the variance, respectively, totaling 53.15%. Lung samples mostly clustered in the first quadrant. Intestinal samples were in the first and fourth quadrants and were more dispersed, indicating greater within-group variability. Other visceral samples were close to lung samples, suggesting similar viral compositions. PERMANOVA analysis showed no significant differences among the three tissue types (*R*^2^ = 0.078, *P* = 0.195).

**Figure 5:**
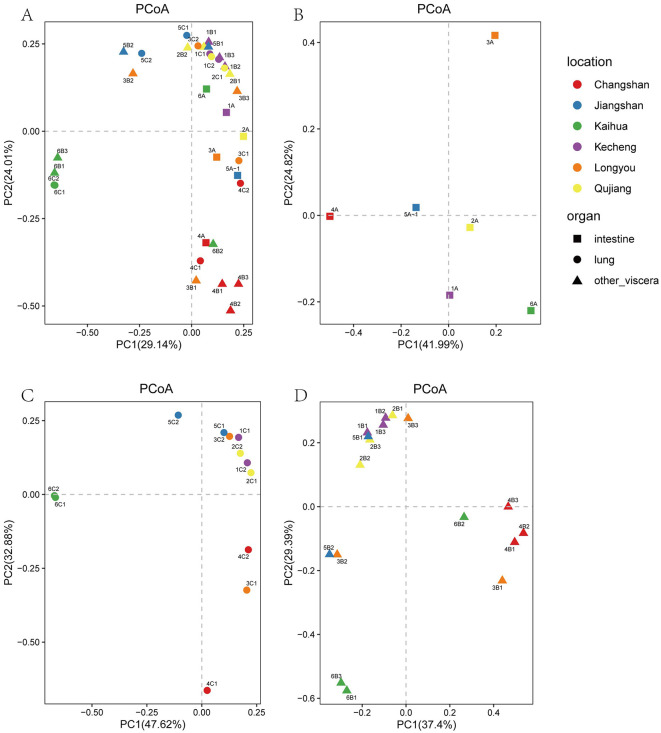
PCoA of pooled libraries grouped by geographic region.

PCoA of viral beta diversity across geographic locations is shown in Figure 5A. PC1 and PC2 explained 29.14 and 24.01% of total variation, respectively. Samples from Kaihua and Changshan Counties were well-separated from those in other regions, while those from Kecheng and Qujiang Districts were more similar. Samples from Jiangshan and Longyou showed greater intra-group differences. PERMANOVA revealed significant differences in viral composition among geographic locations (*R*^2^ = 0.315, *P* < 0.001).

When tissue categories were examined separately by region, intestinal pooled libraries (Figure 5B) showed 66.81% total variance explained (PC1 = 41.99%, PC2 = 24.82%), suggesting substantial regional differentiation. Lung pooled libraries (Figure 5C) showed even stronger regional separation (PC1 = 47.62%, PC2 = 32.88%, total = 80.50%), and the regional effect remained significant by PERMANOVA (*R*^2^ = 0.778, *P* = 0.015). For other visceral pooled libraries (Figure 5D), PC1 and PC2 explained 37.40 and 29.39% of the variance (total = 66.79%), and PERMANOVA again supported a significant regional effect (*R*^2^ = 0.452, *P* = 0.031).

### Phylogenetic analysis of viral contigs

A total of 11 *Coronavirus*-related contigs were detected (Supplementary Figure S1), including four from intestinal samples, four from other visceral samples, and three from lung samples. These were collected from Longyou (3), Kecheng (2), Jiangshan (2), Kaihua (2), Changshan (1), and Qujiang (1). Phylogenetic analysis showed that two contigs belonged to *Alphacoronavirus* (bootstrap = 98), two to *Deltacoronavirus* (bootstrap = 99), six to *Betacoronavirus* (bootstrap = 100), and one to *Gammacoronavirus* (bootstrap = 100).

A total of 22 contigs related to *Flaviviridae* were identified (Supplementary Figure S2): 14 from other visceral samples, four from intestinal samples, and four from lung samples. The samples were mainly from Kaihua (13), Jiangshan (4), Longyou (3) and Changshan (2). Phylogenetic analysis revealed 15 contigs in *Pestivirus*, six in *Hepacivirus*, and one in *Orthoflavivirus*.

Seven *Astroviridae*-related contigs were identified (Supplementary Figure S3): four from intestinal samples, two from other visceral samples, and one from lung samples. These were collected from Kaihua (4), Qujiang (1), Changshan (1), and Jiangshan (1). Phylogenetic analysis showed that two sequences belonged to *Avastrovirus*, and five to *Mamastrovirus*.

Nineteen *Hantaviridae*-related contigs were identified (Supplementary Figure S4): 10 from other visceral samples, eight from lung samples, and one from intestinal samples. Collection sites included Kaihua (10), Longyou (4), Qujiang (3), and Kecheng (2). Phylogenetic analysis showed 10 contigs belonged to *Actinovirus*, five to *Percilovirus*, and 4 to *Agnathovirus*.

Five *Sedoreoviridae*-related contigs were identified (Supplementary Figure S5), all from intestinal samples collected in Kaihua. Phylogenetic analysis indicated that all 5 sequences belonged to the *Mimoreovirus* genus.

Twenty-eight contigs related to *Arenaviridae* were detected (Supplementary Figure S6): 13 from other visceral samples, 11 from lung samples, and four from intestinal samples. These were mostly from Kaihua (11), Longyou (5), Jiangshan (5), Qujiang (4), and Kecheng (3). Phylogenetic analysis showed five contigs in the *Innmovirus* clade, four in *Hartmanivirus*, two in *Antennavirus*, and one in *Reptarenavirus*. Sixteen contigs formed independent branches, suggesting the presence of potentially novel *Arenaviridae* viruses.

Eight *Hepeviridae*-related contigs were (Supplementary Figure S7): five from intestinal samples, two from other visceral samples, and one from lung samples. Collection sites included Kaihua (4), Changshan (2), Qujiang (1), and Jiangshan (1). Phylogenetic analysis based on full-genome sequences from ICTV indicated that all sequences belonged to the *Avihepevirus* clade (bootstrap = 100).

## Discussion

This study provides a first metatranscriptomic overview of bat-associated RNA viruses detected in pooled tissues from bats sampled across six administrative areas of Quzhou City. Seven RNA viral families were identified, and regional structure in viral composition was evident across pooled libraries. At the same time, the study design requires cautious interpretation. The data are derived from pooled tissues, not from individual bats; sequence-based detection does not establish host association; and host species and geographic region were fully confounded because *Hipposideros armiger* was sampled only in Kecheng, Qujiang, and Jiangshan, whereas *Rhinolophus sinicus* was sampled only in Longyou, Changshan, and Kaihua. Species-level ecological inferences are therefore not warranted from the present dataset.

*Coronaviridae* was the most widespread family detected in pooled libraries, which is consistent with the broad diversity, recombination potential, and genomic plasticity reported for *coronaviruses* (17, 18). *Flaviviridae* was the second most frequent family. The prominence of *flaviviral* contigs in some pooled libraries may reflect ecological exposure opportunities in a subtropical region with abundant arthropods during the sampling season (19–23), but the present data do not resolve transmission routes or reservoir status. By contrast, *Sedoreoviridae* and *Arenaviridae* were less frequently represented, which may reflect lower abundance, uneven regional occurrence, or limitations of pooled sampling (24).

The most robust community-level finding in this study is the significant regional structuring of viral composition. Regional effects were supported in the overall data set and remained evident in lung and other-visceral pooled libraries. However, the environmental drivers of these differences were not directly measured. Differences in topography, rainfall, vegetation, roost characteristics, host population connectivity, arthropod communities, or local wildlife assemblages could all contribute, but these explanations remain hypotheses for future investigation rather than demonstrated mechanisms in the present study (17). Although Figure 3 displays a species-based ordination, the complete confounding between host species and region means that it should not be interpreted as evidence of species-specific viral community differences.

The *coronavirus*-related contigs identified here document genetic diversity within *alpha*-, *beta*-, *delta*-, and *gammacoronavirus* lineages in pooled bat-associated samples from Quzhou. In particular, six contigs grouped with *Betacoronavirus*. However, because these detections are based on partial contigs from pooled libraries, without complete genomes, without spike-gene resolution, and without independent validation by Reverse Transcription-Polymerase Chain Reaction/Real-time Quantitative Polymerase Chain Reaction (RT-PCR/qPCR), Sanger sequencing, or virus isolation, they should not be interpreted as evidence of pathogenicity, zoonotic potential, or public-health risk (25). We therefore restrict our interpretation to the presence of *betacoronavirus*-like sequence diversity in the pooled samples analyzed here. Likewise, the occurrence of *Deltacoronavirus*-related contigs may reflect ecological overlap with other vertebrate hosts in the region, but the present data do not establish cross-species transmission.

For *Flaviviridae*, most sequences grouped with *Pestivirus* and *Hepacivirus*. Because members of these groups have been reported from diverse wildlife hosts (26–28), the present detections add to the known sequence diversity associated with bat-containing sample pools. Nonetheless, pooled-tissue sequence recovery alone cannot determine whether the bat was the biological host, an incidental carrier, or a source of diet- or environment-derived viral material. Similar caution applies to *Astroviridae*. The *Mamastrovirus*-related contigs and the two *Avastrovirus*-related contigs indicate phylogenetic diversity, but they do not establish natural host range. The genetically divergent *Mamastrovirus*-like sequences are best interpreted as candidate lineages requiring validation, genome completion, and host-association analysis (29–32).

The *Hantaviridae* results warrant particular caution. Three of the detected genera—*Actinovirus, Agnathovirus*, and *Percilovirus*—are currently associated in ICTV taxonomy with fish hosts. Their recovery from pooled bat tissues is therefore taxonomically incongruous and may reflect bioinformatic misassignment, environmental contamination, indirect dietary input, or other non-host-associated explanations. Because of this uncertainty, we do not interpret these detections as evidence that bats in Quzhou are reservoirs of these *hantavirid* lineages, and we do not link them to local hemorrhagic fever with renal syndrome (HFRS) incidence data.

The five *Sedoreoviridae*-related contigs all grouped within *Mimoreovirus* and were detected only in an intestinal pooled library from Kaihua. Given the restricted detection pattern and the limited understanding of *mimoreovirus* ecology (33), these sequences are described as *Mimoreovirus*-related contigs detected in pooled bat-associated intestinal material. Their biological origin, tissue association, and epidemiological relevance remain unclear.

*Arenaviridae* showed substantial phylogenetic diversity in this study. Several contigs grouped with recognized *arenavirid* clades, whereas 16 formed independent branches. These findings suggest that bats or bat-associated environments in Quzhou contain genetically diverse *arenavirid*-like sequences. However, the biological and public-health relevance of these lineages remains uncertain and requires additional validation, including recovery of longer genomic regions, confirmation in individual specimens, and comparison with known *mammarenavirus* diversity (34, 35).

The *Hepeviridae* findings also require a restrained interpretation. All eight *hepevirid* contigs grouped within *Avihepevirus*, a lineage generally regarded as bird-associated. Because both bat species in the present study are strictly insectivorous and do not prey on birds, a conventional dietary explanation through predation on birds is implausible. More plausible explanations include cave-environment contamination, indirect exposure through shared habitats, or taxonomic assignment uncertainty. Accordingly, we do not interpret the present data as evidence that these bats are natural hosts of *Avihepevirus*, and the host range implications of these contigs should remain open pending validation (36).

This study has several limitations. First, the organ- and geography-based pooling strategy improved throughput and virus-discovery efficiency but reduced per-individual resolution and may have diluted low-titer viruses. The multiple pooled libraries within each region and tissue category should be viewed as independently prepared pooled libraries rather than technical replicates, and they do not substitute for individual-level sampling. Second, the study relied on assembly- and homology-based bioinformatic pipelines and did not include independent laboratory validation or virus isolation. Third, host species and geographic region were fully confounded, preventing independent interpretation of species vs. region effects. Finally, environmental variables that could explain regional structuring were not directly measured.

## Conclusion

In this exploratory metatranscriptomic survey, pooled tissue libraries from bats sampled across six administrative areas of Quzhou yielded viral contigs assigned to seven RNA viral families: *Coronaviridae, Flaviviridae, Astroviridae, Hepeviridae, Hantaviridae, Sedoreoviridae*, and *Arenaviridae*. *Coronaviridae* was the most frequently detected family, and viral community composition differed significantly among regions. The intestinal pooled library from Kecheng District showed the greatest family-level diversity. However, because the study was based on pooled samples, sequence homology, and phylogenetic placement without independent validation, the findings should be interpreted as baseline evidence of viral diversity and geographic structuring rather than confirmation of host association, zoonotic potential, or transmission risk. Future work should prioritize sequence deposition, targeted molecular confirmation, genome completion, individual-level sampling, and ecological measurements to clarify the biological relevance of the detected viral lineages.

## Data Availability

The data presented in the study are deposited in the NCBI Sequence Read Archive (SRA) under BioProject accession number PRJNA1474706.
