# Supplementary material for: Exploratory metatranscriptomic survey of bat-associated RNA viruses in Quzhou city, China
Source: Front Vet Sci. 2026 Jul 10;13:1721079. doi: 10.3389/fvets.2026.1721079 (PMC13395664; doi:10.3389/fvets.2026.1721079)
Supplement: Supplementary file 1 [file Data_Sheet_1.zip › Supplementary material 1.docx]

**1. Bat Species Identification**

**1.1 Tissue Homogenization**

Bat tissue samples were rinsed with phosphate-buffered saline (PBS) and placed in a glass homogenizer. Then, 600 μL of PBS buffer was added, and the tissues were thoroughly ground into a homogeneous slurry.

**1.2 Total DNA Extraction**

DNA was extracted using a commercial kit following the manufacturer’s instructions. The main steps were as follows:

(1) 20 μL of Proteinase K was added to the homogenate, followed by incubation at 56 °C for 3 minutes. The mixture was vortexed every 15 seconds during incubation.

(2) 200 μL of AL buffer was added and mixed thoroughly, then incubated at 56 °C for 10 minutes.

(3) 200 μL of absolute ethanol was added to the mixture.

(4) The mixture was transferred to a DNA adsorption column placed in a collection tube and centrifuged at 9000 rpm for 1 minute. The flow-through was discarded.

(5) 500 μL of AW1 buffer was added to the column, followed by centrifugation at 8000 rpm for 1 minute. The flow-through and collection tube were discarded.

(6) 500 μL of AW2 buffer was added, and the column was centrifuged at 14,000 rpm for 3 minutes. Again, the flow-through and collection tube were discarded.

(7) The DNA was eluted with 50 μL of Buffer AE through centrifugation at 8000 rpm for 1 minute. This elution step was repeated once to increase yield.

(8) The extracted DNA was stored in sealed collection tubes. A 1 μL aliquot was taken to assess DNA concentration and integrity. The remaining DNA was stored at –20 °C.

**1.3 PCR Amplification**

Bat species were molecularly identified by amplifying the mitochondrial cytochrome b (Cytb) gene via polymerase chain reaction (PCR). Primer sequences are listed in Supplementary Table 1, and the PCR reaction setup is provided in Supplementary Table 2.

Table S1 Primer sequences of bat species

| Name | Sequence | Fragment size |
| --- | --- | --- |
| Bat_Cytb_R | GGAGGAAGTGCAGGGCRAARAATCG | 559bp |
| Bat_Cytb_F | ATGACCAACATYCGIAAATCHCAYCC |  |

Table S2 amplification reaction system

| Component | Dosage（μL） |
| --- | --- |
| 5×TransStart® FastPfu Buffer | 5 |
| dNTPs | 2 |
| DNA | 5 |
| F/R | 1/1 |
| TransStart(®) FastPfu DNA Polymerase | 0.5 |
| ddH2O | 25 |

**1.4 PCR Product Verification and Sequencing**

A 2 μL aliquot of the PCR product was separated by 1% agarose gel electrophoresis. The target band was excised and gel-purified. The purified product was sent to Shanghai BioGerm Medical Biotechnology Co., Ltd. for Sanger sequencing to confirm bat species identification.

**2. Nucleic Acid Extraction**

**2.1 Pre-processing**

A soybean-sized tissue sample was taken and homogenized in 200 μL of PBS buffer until a slurry was obtained.

**2.2 RNA Extraction**

RNA was extracted using the QIAGEN AllPrep Power Viral RNA Kit according to the manufacturer’s protocol. The procedure was performed as follows:

(1) 200 μL of the pre-processed sample was mixed with 600 μL of PM1 buffer containing β-mercaptoethanol (B-ME) in a Power Bead Tube. The mixture was processed using a high-throughput tissue homogenizer under the following conditions: 6 m/s, 120 s per cycle, 30 s pause, repeated for 5 cycles.

(2) The Power Bead Tube was centrifuged at 13,000 × g for 1 minute. The supernatant was transferred to a new tube and mixed with 150 μL of IRS solution, followed by incubation at room temperature for 5 minutes.

(3) The mixture was centrifuged at 13,000 × g for 1 minute. Then, 700 μL of supernatant was taken and mixed with an equal volume (600 μL) of a PM3 and PM4 solution mixture. A total of 625 μL of this mixture was loaded onto an MB Spin Column and centrifuged at 13,000 × g for 1 minute. The flow-through was discarded. This step was repeated until all the supernatant had passed through the column.

(4) The column was washed with 600 μL of PM5 buffer and centrifuged at 13,000 × g for 1 minute. The flow-through was discarded. This was followed by a wash with 600 μL of PM4 buffer and another centrifugation under the same conditions. Finally, the column was centrifuged at 13,000 × g for 2 minutes to remove any residual liquid.

(5) The MB Spin Column was transferred to a new collection tube. Then, 100 μL of nuclease-free water was added to the column matrix and incubated at room temperature for 3 minutes. RNA was eluted by centrifugation at 13,000 × g for 1 minute, and the resulting RNA solution was collected.

**2.3 Nucleic Acid Quality Control**

(1) A 2 μL aliquot of the extracted RNA was quantified using a spectrophotometer.

(2) Samples meeting quality standards were used for subsequent library construction.

Table 3 sample nucleic acid quality control results

| Sample number | Fluorescence concentration (ng/μL) | total (ng) | Sample number | Fluorescence concentration (ng/μL) | total (ng) |
| --- | --- | --- | --- | --- | --- |
| 1A | 577.513 | 28875.65 | 3B3 | 382.476 | 19123.8 |
| 2A | 586.163 | 58616.3 | 3C1 | 428.348 | 21417.4 |
| 3A | 137.292 | 13729.2 | 3C2 | 495.227 | 24761.35 |
| 4A | 830.721 | 83072.1 | 4B1 | 769.489 | 38474.45 |
| 5A-1 | 163.205 | 16320.5 | 4B2 | 818.177 | 40908.85 |
| 6A | 264.967 | 26496.7 | 4B3 | 768.187 | 38409.35 |
| 1B1 | 367.125 | 18356.25 | 4C1 | 518.4 | 25920 |
| 1B2 | 575.741 | 28787.05 | 4C2 | 152.711 | 7635.55 |
| 1B3 | 246.643 | 12332.15 | 5B1 | 547.583 | 27379.15 |
| 1C1 | 678.404 | 33920.2 | 5B2 | 396.738 | 19836.9 |
| 1C2 | 734.667 | 36733.35 | 5C1 | 493.539 | 24676.95 |
| 2B1 | 464.309 | 23215.45 | 5C2 | 387.977 | 19398.85 |
| 2B2 | 546.349 | 27317.45 | 6B1 | 382.498 | 19124.9 |
| 2B3 | 1068.194 | 53409.7 | 6B2 | 274.165 | 13708.25 |
| 2C1 | 392.709 | 19635.45 | 6B3 | 633.479 | 31673.95 |
| 2C2 | 484.426 | 24221.3 | 6C1 | 289.055 | 14452.75 |
| 3B1 | 581.325 | 29066.25 | 6C2 | 706.415 | 35320.75 |
| 3B2 | 381.121 | 19056.05 |  |  |  |

**3. RNA Transcriptome Library Construction**

**3.1 Depletion of Prokaryotic rRNA**

**3.1.1 Probe Hybridization**

(1) Thaw the probes, streptavidin magnetic beads, and hybridization buffer. Mix the probes thoroughly and keep them on ice. All other reagents can be used at room temperature.

(2) Dilute the RNA sample to 12 μL using nuclease-free H₂O.

(3) Prepare the rRNA depletion reaction mixture according to the ratios listed in Supplementary Table 4.

Table S4 rRNA removal reaction system

| Component | Dosage（μL） |
| --- | --- |
| Total RNA | 12（10 ng~1 μg） |
| Hybridization Buffer | 5 |
| Probe Mix | 3 |

(4) Centrifuge the mixture briefly to collect all liquid at the bottom of the tube.

(5) Place the PCR tube in a thermal cycler and run the probe hybridization reaction using the program specified in Supplementary Table 5.

Table S5 probe hybridization procedure

| Temperature | Time |
| --- | --- |
| Heated lid: 105℃ | On |
| 68℃ | 10 min |
| 37℃ | 0.1℃/s |
| 37℃ | 2 min |

**3.1.2 Preparation of Streptavidin Magnetic Beads**

(1) Resuspend the streptavidin magnetic beads by vortexing. After mixing and brief centrifugation, transfer 40 μL to a new tube. Let the tube stand for 2 minutes, then discard the supernatant.

(2) Add 80 μL of Depletion Buffer, mix thoroughly, and incubate for 2 minutes. Discard the supernatant. Repeat this step once.

**3.1.3 rRNA Depletion**

(1) Centrifuge the hybridized sample from step 3.1.1 to collect all liquid at the bottom of the tube.

(2) Add the prepared magnetic beads from step 3.1.2 to the sample. Mix well and place the tube in a thermal cycler. Perform probe capture using the program detailed in Supplementary Table 6.

Table S6 rRNA Depletion Program

| Temperature | Time |
| --- | --- |
| Heated lid: 105℃ | On |
| 37℃ | 15 min |
| 50℃ | 5 min |

(3) Centrifuge the product and let it stand for 2 minutes. Transfer the supernatant to a new tube.

**3.1.4 RNA Purification**

(1) Take Purification Beads and allow them to equilibrate at room temperature for 30 minutes. Prepare an 80% ethanol solution.

(2) Mix the beads thoroughly, add 180 μL of the sample, and incubate at room temperature for 5 minutes.

(3) Place the tube on a magnetic stand until the solution clears. Discard the supernatant.
(4) Wash the beads twice with 80% ethanol, incubating for 30 seconds each time before discarding the supernatant.

(5) Air-dry the beads for 5–10 minutes. Add 11 μL of nuclease-free water, mix, and incubate for 5 minutes to elute the RNA.

(6) Centrifuge the tube and let it stand for 3 minutes. Transfer 10 μL of the supernatant to a new tube.

**3.2 RNA Fragmentation**

(1) Measure the RNA concentration using a NanoDrop spectrophotometer.

(2) Prepare a premix containing 8.5 μL of RNA sample and Frag/Prime Buffer. Perform the RNA fragmentation reaction.

**3.3 Double-Stranded cDNA Synthesis**

(1) Thaw all required reagents on ice. Mix and centrifuge briefly. Prepare the first-strand cDNA synthesis reaction mixture according to Supplementary Table 7.

Table S7 First-Strand cDNA Synthesis Reaction System

| Component | Dosage（μL） |
| --- | --- |
| Fragmented mRNA | 17 |
| Strand Specificity Reagent | 3 |
| 1st Strand Enzyme Mix | 2 |

(2) Mix well and perform first-strand cDNA synthesis in a thermal cycler.

(3) Prepare the second-strand cDNA synthesis reaction mixture as described in Supplementary Table 8.

Table S8 Second-Strand cDNA Synthesis Reaction System

| Component | Dosage（μL） |
| --- | --- |
| 1st Strand cDNA | 25 |
| 2nd Strand Buffer 2 | 30 |
| 2nd Strand Enzyme Master Mix | 5 |

(4) Mix thoroughly and carry out second-strand cDNA synthesis in a thermal cycler.

**3.4 Adapter Ligation**

(1) Prepare the adapter ligation reaction mixture following the formulation in Supplementary Table 9.

Table S9 Adapter Ligation Reaction System

| Component | Dosage（μL） |
| --- | --- |
| ds cDNA | 60 |
| Ligation Enhancer | 30 |
| DNA Adapter | 5 |
| Novel T4 DNA Ligase | 5 |

(2) Mix gently and perform the ligation reaction using the following program: 20 °C for 15 minutes, then hold at 4 °C.

**3.5 Product Purification**

(1) Mix VAHTS DNA Clean Beads thoroughly. Add 25 μL of beads to the ligation product, mix well, and incubate at room temperature for 5 minutes.

(2) Place the tube on a magnetic stand. Transfer the supernatant to a new tube, add 10 μL of clean beads, incubate, and discard the supernatant.

(3) Wash the beads twice with 80% ethanol, incubating for 30 seconds each time before discarding the supernatant.

(4) Air-dry the beads for 5–10 minutes. Add 22.5 μL of nuclease-free water, mix, and incubate for 2 minutes.

(5) Place the tube on a magnetic stand. Transfer 20 μL of the supernatant to a new PCR tube.

**3.6 Library Amplification**

(1) Prepare the PCR amplification reaction mixture as outlined in Supplementary Table 10.

Table S10 PCR Amplification Reaction System

| Component | Dosage（μL） |
| --- | --- |
| Product | 20 |
| 2×SuperCanace®IIHigh-FidelityMix | 25 |
| Index Primer/i7Primer | 2.5 |
| Universal Primer/i5Primer | 2.5 |

(2) Mix thoroughly and perform library amplification in a thermal cycler using the program specified in Supplementary Table 11.

Table S11 Library Amplification Program

| **Program** | Temperature | Time | Cycles |
| --- | --- | --- | --- |
| Pre-denaturation | 98℃ | 1 min | 1 |
| Denaturation | 98℃ | 10 s | 16 |
| Annealing | 60℃ | 30 s |  |
| Extension | 72℃ | 30 s |  |
| Final Extension | 72℃ | 5 min | 1 |
| Hold | 4℃ | ∞ | 1 |

**3.7 Library Purification**

(1) Allow magnetic beads to reach room temperature. Add the entire reaction system to the beads, mix thoroughly, and incubate at room temperature for 5 minutes.

(2) Place the tube on a magnetic stand until the solution becomes clear. Carefully discard the supernatant.

(3) Wash the beads twice with 80% ethanol, incubating for 30 seconds each time before completely removing the supernatant.

(4) Air-dry the beads at room temperature for 5–10 minutes. Add 32 μL of nuclease-free water, mix well, and incubate for 5 minutes to elute the DNA.

(5) Place the tube on the magnetic stand until the solution clears. Transfer 30 μL of the supernatant to a new PCR tube.

(6) Dilute 2 μL of the purified library in 198 μL of the working solution from the dsDNA HS Assay Kit for Qubit. Mix gently and incubate in the dark for 2 minutes. Measure the library concentration using a Qubit fluorometer, ensuring the total amount exceeds 20 ng.

**4. Library Quality Control**

Library concentration was accurately quantified using a Qubit 3.0 Fluorometer. The size distribution and integrity of the library fragments were assessed using an Agilent 2100 Bioanalyzer to confirm they met the expected specifications (e.g., predominant peak within the desired size range). Libraries passing quality control were pooled in equimolar ratios according to sequencing requirements for subsequent preparation.

Table S12 library quality control results

| Sample name | Qubit (ng/μL) | Average fragment size（bp） | Sample name | Qubit (ng/μL) | Average fragment size（bp） |
| --- | --- | --- | --- | --- | --- |
| 1B2、1B3、2B1 | 20.2 | 361.0 | 1A | 8.5 | 371.3 |
| 2B2、2B3、3B1 | 24.2 | 369.7 | 2A | 9.64 | 407.0 |
| 3B2、3B3、4B1 | 11.8 | 350.2 | 3A | 13.6 | 387.0 |
| 1C2、4C1、4C2 | 25.2 | 407.4 | 4A | 8.78 | 375.3 |
| 6B1、6B2、6B3 | 18.6 | 332.1 | 5A | 11.6 | 361.5 |
| 1B1、1C1 | 8.5 | 371.3 | 6A | 11.8 | 393.0 |
| 4B2、4B3 | 13.6 | 346.0 | 3C1、3C2 | 25.2 | 407.4 |
| 5B1、5B2 | 13.6 | 346.0 | 5C1、5C2 | 25.2 | 407.4 |
| 6C1、6C2 | 25.2 | 407.4 | 2C1、2C2 | 25.2 | 407.4 |

**5. Next-Generation Sequencing**

Qualified libraries were subjected to paired-end 150 bp (PE150) sequencing on the DNBSEQ-T7 platform. The sequencing procedure consisted of the following steps: Genomic DNA was fragmented, end-repaired using Klenow fragment, and ligated with adapters. DNA nanoballs (DNBs) were generated through rolling circle amplification. The DNBs were then loaded onto a sequencing chip. Sequencing was performed using combinatorial probe-anchor synthesis (cPAS) technology. Fluorescently labeled dNTPs were incorporated based on the principle of sequencing-by-synthesis. Fluorescence signals were captured in real-time and computationally converted into nucleotide sequences for downstream bioinformatic analysis.

**4. Bioinformatics Software**

Table S13. Bioinformatics Software and Applications

| Software | Version | Purpose |
| --- | --- | --- |
| Kraken2 | v2.1.2 | Read-based taxonomic classification |
| MEGAHIT | v1.2.9 | De novo assembly of short reads |
| DIAMOND | v2.1.8.162 | Fast sequence alignment and search |
| fastp | v0.23.2 | Quality control and preprocessing of reads |
| Samtools | v0.1.19 | Processing and handling SAM/BAM files |
| IQ-TREE | v2.0.3 | Phylogenetic tree reconstruction |
| Mafft | v7.515 | Multiple sequence alignment |
| ITOL | v7 | Visualization and annotation of phylogenetic trees |
| seqkit | v2.2.0 | Efficient processing of FASTA/Q sequences |
| R | v4.2.2 | Statistical analysis and data visualization |

**6. Databases**

Table S14. Databases and Their Applications

| Database | URL | Purpose |
| --- | --- | --- |
| ICTV | https://ictv.global/ | Virus nomenclature and classification |
| NCBI RefSeq | https://ftp.ncbi.nlm.nih.gov/blast/db/ | Reference sequences for annotation and BLAST |
