# Supplementary material for: Exploratory metatranscriptomic survey of bat-associated RNA viruses in Quzhou city, China
Source: Front Vet Sci. 2026 Jul 10;13:1721079. doi: 10.3389/fvets.2026.1721079 (PMC13395664; doi:10.3389/fvets.2026.1721079)
Supplement: Supplementary file 1 [file Data_Sheet_1.zip › Supplementary material 2.docx]

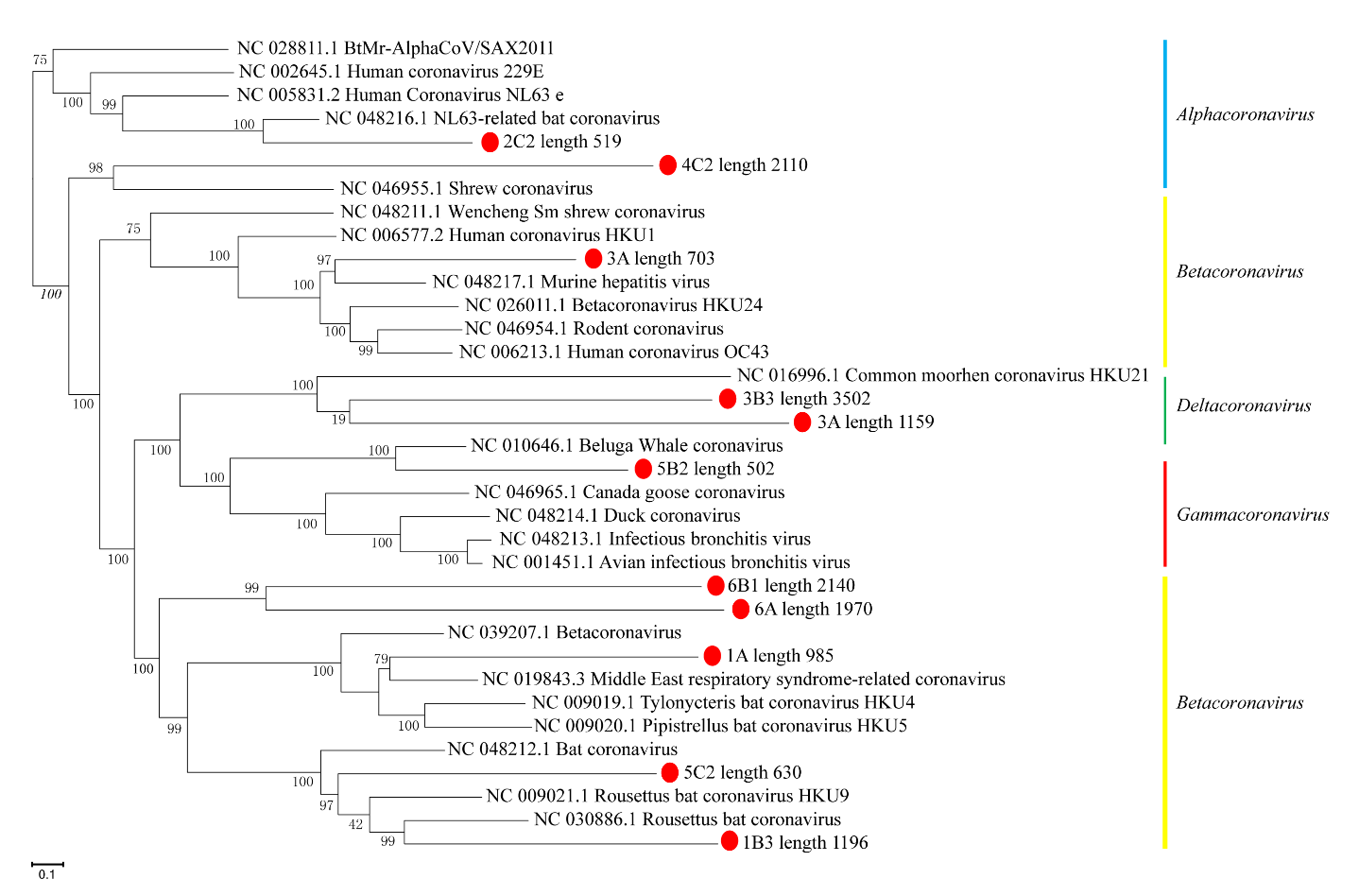
Supplementary Figure S1. Phylogenetic analysis of Coronaviridae


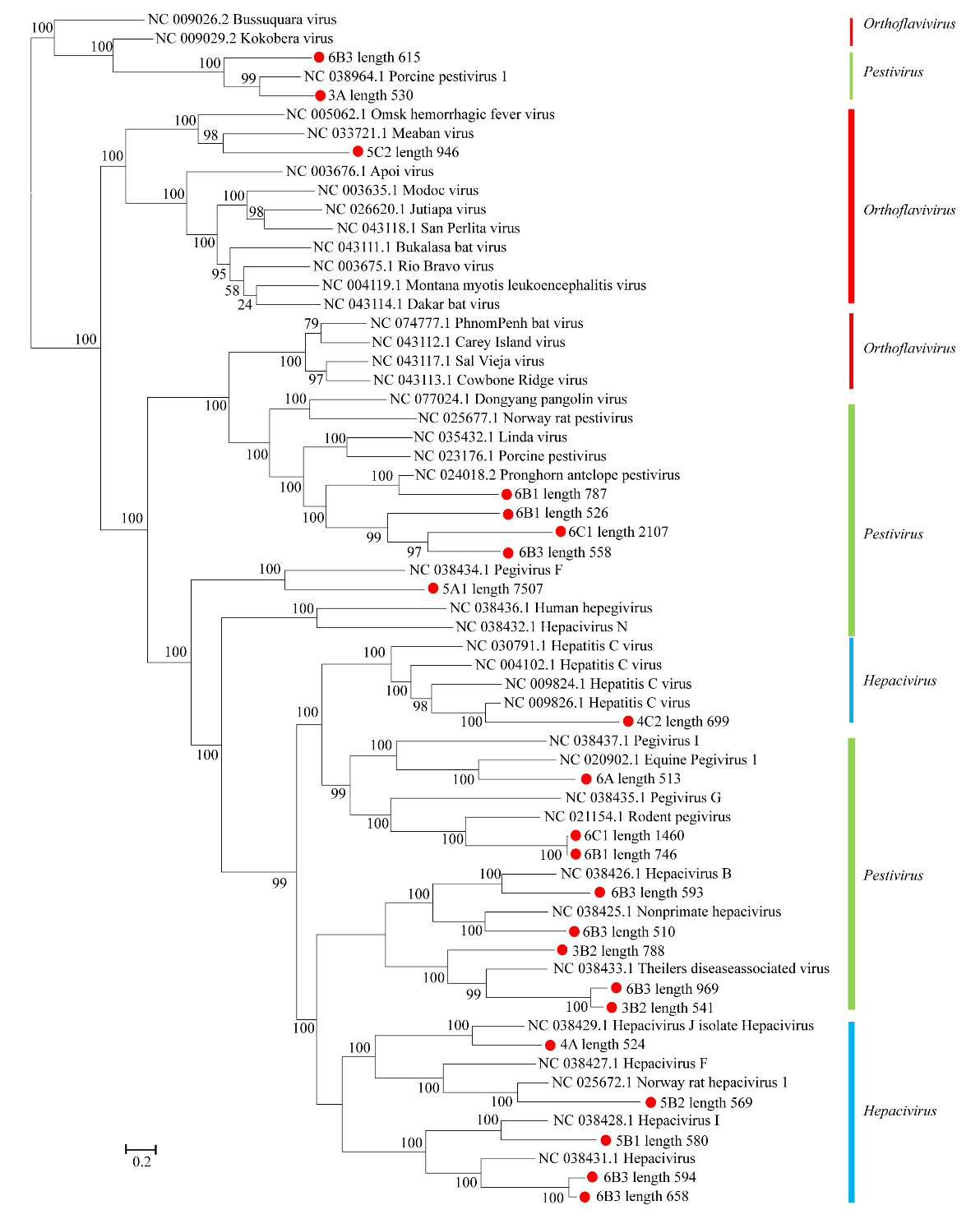


Supplementary Figure S2. Phylogenetic analysis of Flaviviridae


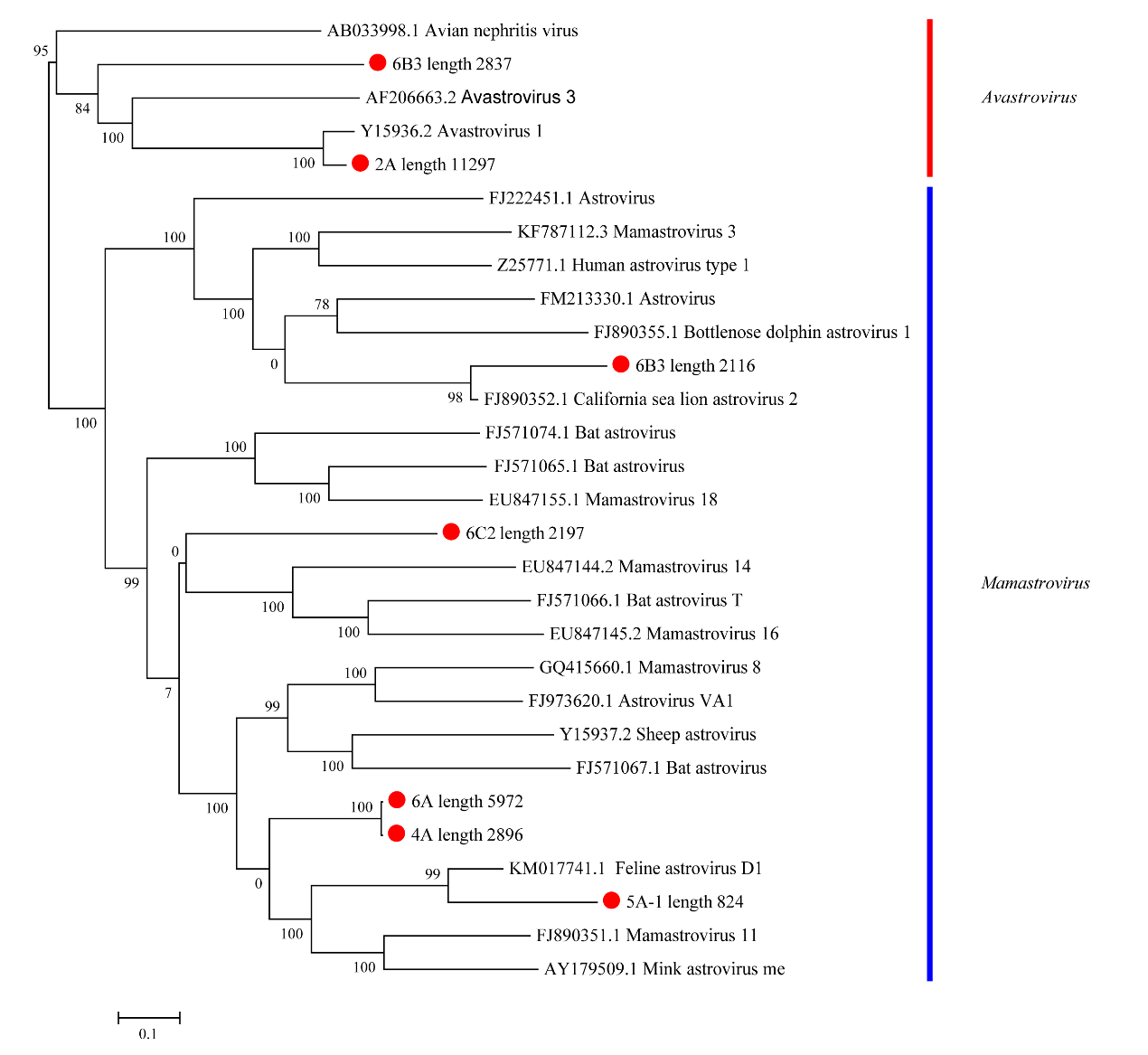


Supplementary Figure S3. Phylogenetic analysis of Astroviridae


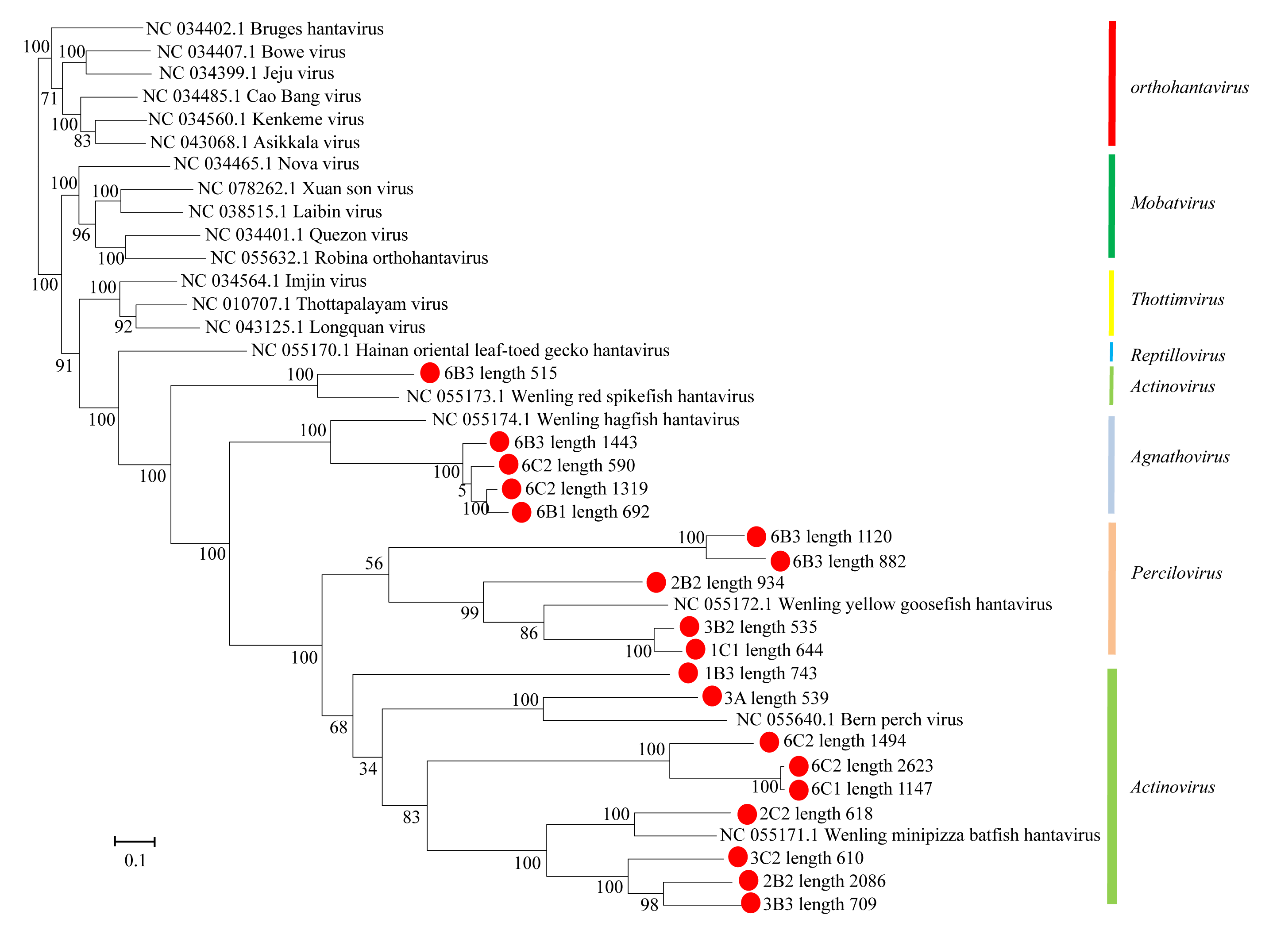


Supplementary Figure S4. Phylogenetic analysis of Hantaviridae


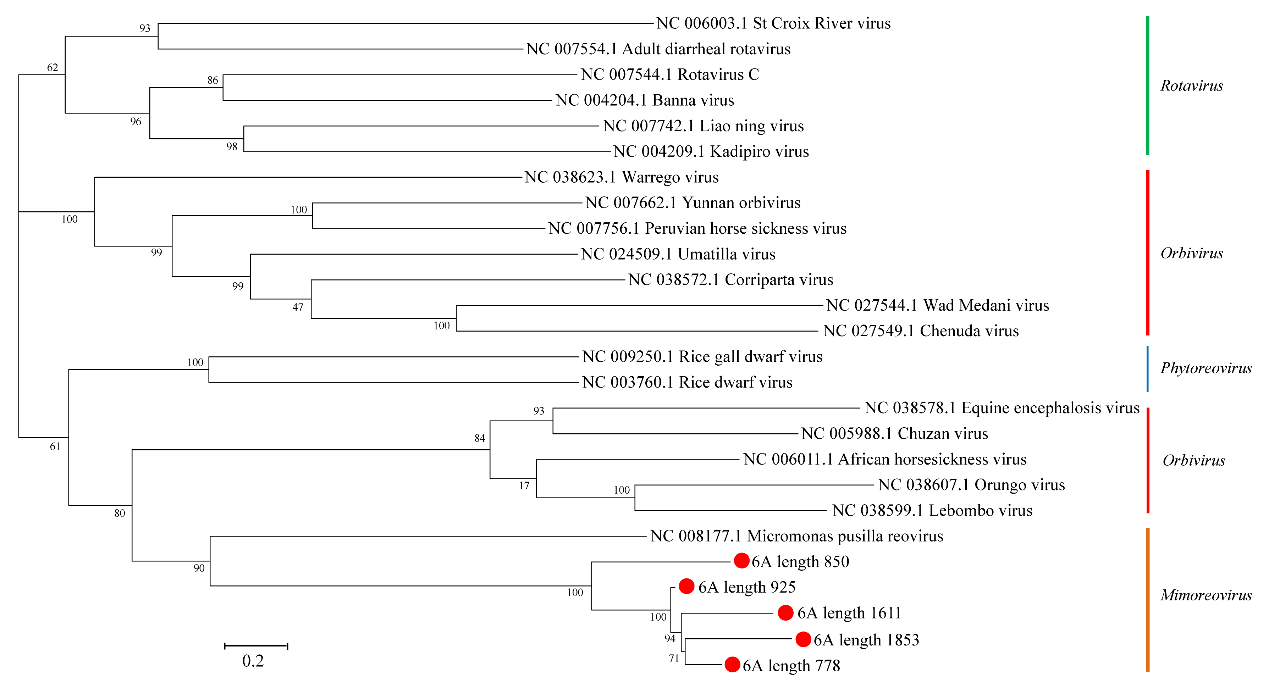


Supplementary Figure S5. Phylogenetic analysis of Sedoreoviridae


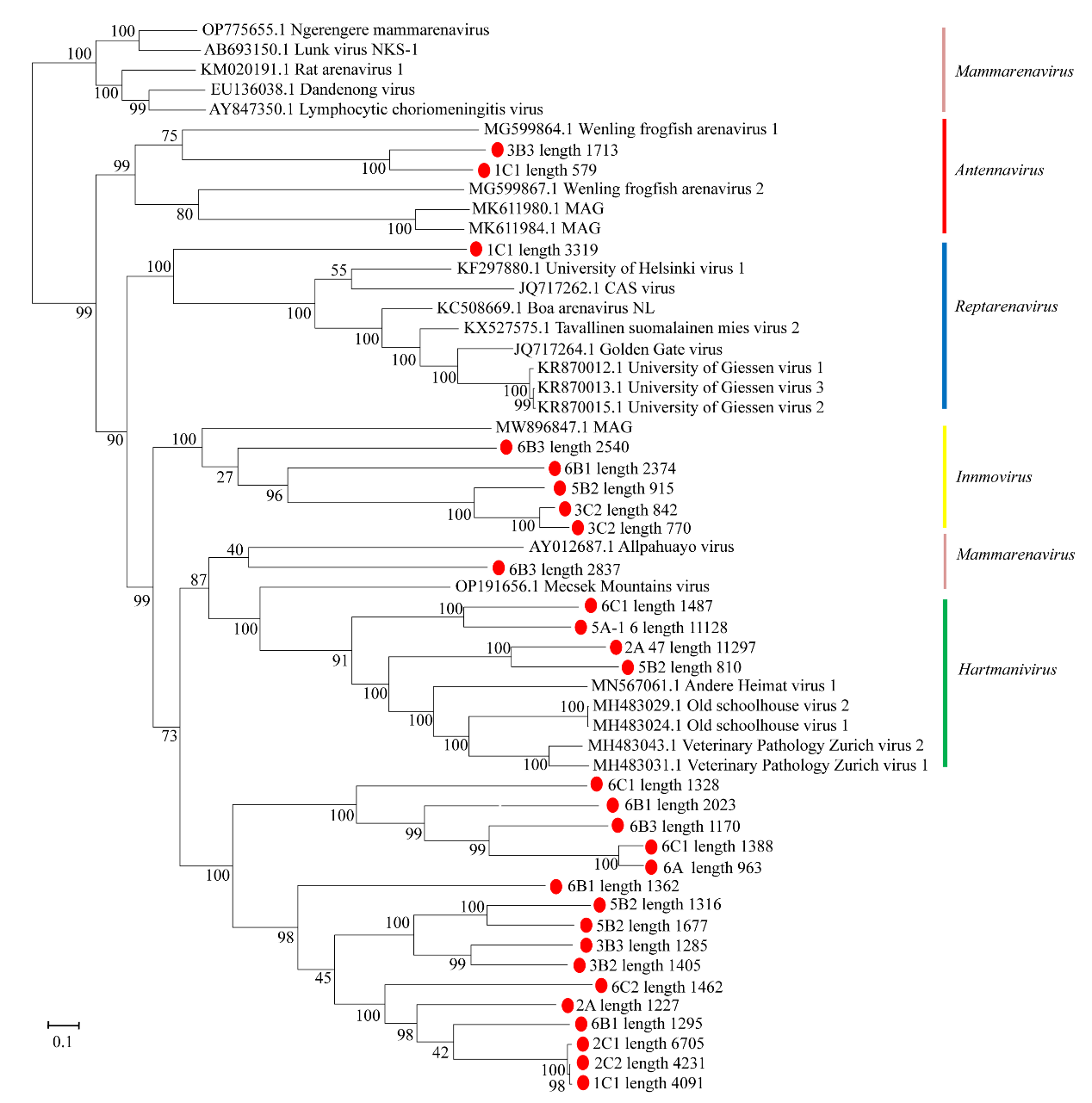


Supplementary Figure S6. Phylogenetic analysis of Arenaviridae


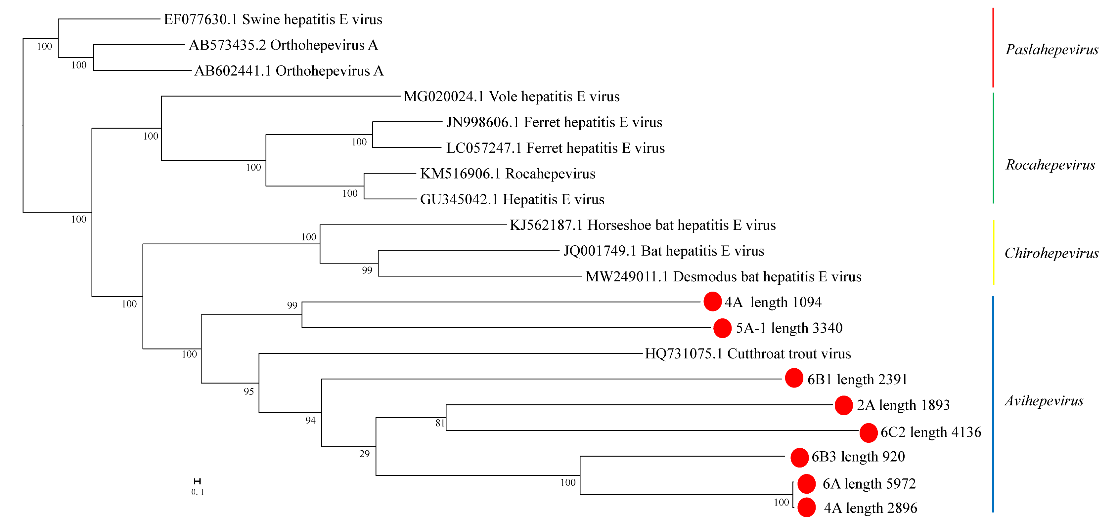


Supplementary Figure S7. Phylogenetic analysis of Hepeviridae
